# Patients Having Major Abdominal Cancer Surgery Exhibit Significant Acute Muscle Wasting

**DOI:** 10.1002/jcsm.13858

**Published:** 2025-06-13

**Authors:** Gabor Dudas, Ismita Chhetri, Martin Whyte, Margaret Rayman, Rachel Simmonds, Anthony Catchpole, Mark Griffiths, Ben Creagh‐Brown

**Affiliations:** ^1^ Faculty of Health and Medical Sciences University of Surrey Guildford UK; ^2^ Intensive Care Medicine Royal Surrey NHS Foundation Trust Guildford UK; ^3^ Scottish Trace Element and Micronutrient Diagnostic and Research Laboratory Glasgow Royal Infirmary Glasgow UK; ^4^ William Harvey Institute Queen Mary University of London London UK; ^5^ National Heart and Lung Institute Imperial College London UK; ^6^ Peri‐Operative Medicine, Barts Heart Centre St Bartholomew's Hospital London UK

**Keywords:** cancer surgery, insulin resistance, muscle loss, muscle wasting, myopenia, postoperative recovery

## Abstract

**Background:**

Postoperative myopenia (acute muscle loss) is a significant concern following major cancer resection surgery, contributing to prolonged recovery, increased dependency and impaired quality of life. Despite its clinical relevance, the mechanisms underlying postoperative myopenia and its potential mediators remain poorly understood.

This study aims to evaluate the acute changes in muscle size, strength and activity following major cancer surgery and to explore the role of insulin resistance and selenium deficiency as potential mediators of these alterations.

**Methods:**

A prospective cohort study was conducted involving 52 patients undergoing elective open major abdominal surgery for cancer. Preoperative and postoperative assessments included measurements of rectus femoris cross‐sectional area (RFCSA) via ultrasound, handgrip strength (HGS), sniff nasal inspiratory pressure (SNIP) and physical activity using an accelerometer. Blood samples were analysed for markers of muscle metabolism, systemic inflammation, insulin resistance and selenium levels. Statistical analyses were performed to compare preoperative and postoperative values and to explore correlations between these measures and clinical outcomes.

**Results:**

A significant reduction in RFCSA was observed in 50% of patients, with a median decrease of 10.2% within the first week post‐surgery, which persisted at the 6‐week follow‐up. HGS and SNIP also showed significant declines postoperatively, and reduced physical activity was associated with greater muscle loss. Insulin resistance and postoperative selenium depletion were associated with greater reductions in RFCSA.

**Conclusion:**

Major cancer surgery is associated with significant acute muscle loss, which is not fully recovered by 6 weeks postoperatively. Insulin resistance and selenium deficiency may contribute to this muscle loss. Further research is needed to investigate potential interventions to prevent or mitigate postoperative myopenia.

**Abbreviations**
APACHE II: Acute Physiology and Chronic Health Evaluation II
ARISCAT: Assess Respiratory Risk in Surgical Patients in Catalonia
ICNARC: Intensive Care National Audit & Research Centre
POMS: Postoperative Morbidity Survey
POPC: Postoperative Pulmonary Complications

## 1 Introduction

As the age of the population increases, so does the incidence of cancer, and the number of patients undergoing cancer resection surgery [1]. Recovery of physical function after such operations is often incomplete, even in the absence of recognised complications [2]. Patients with sarcopenia or cachexia prior to surgery are predisposed to adverse outcomes [3], but the acute loss of muscle (myopenia) following surgery is under‐recognised. Myopenia can contribute to incomplete physical recovery, which may lead to increased dependency and impaired quality of life. Interventions to prevent or treat acute muscle loss are lacking [4]. As well as being clinically significant in itself, myopenia after surgery has similarities to loss of muscle associated with critical illness (intensive care unit acquired weakness, ICU‐AW) plausibly including shared pathophysiological mechanisms [4, 5].

Muscle weakness, loss of muscle mass or reduced dimensions of muscles on imaging represents different manifestations of myopenia. Use of ultrasound to measure serially the quadriceps (including the rectus femoris [RF]) [6] has shown promise as a surrogate of functional outcomes in ICU‐AW [7]. Impaired handgrip strength (HGS) was independently associated with mortality in a population with ICU‐AW in many surgical cohorts [8] and indeed in the general population [9]. Postoperative pulmonary complications have multifactorial aetiology, but impaired diaphragm function predisposes to their development [10]. Finally, physical activity monitors have shown that reduced activity after surgery was associated with adverse outcomes [11].

Muscle wasting occurs due to an imbalance between anabolic and catabolic processes [12]. The role of circulating mediators in perioperative muscle wasting is supported by recent findings in muscle biopsies from patients having abdominal surgery, with early changes of muscle inflammation, mitochondrial dysfunction and metabolic dysregulation remote to the site of surgery [13, 14]. Insulin‐like growth factor 1 (IGF‐1) is anabolic, and suppressed levels have been associated with muscle loss in a range of conditions [15]. Two members of the transforming growth factor‐β (TGF‐β) family, myostatin [16] and growth and differentiation factor‐15 (GDF‐15) [17], have been associated with acute loss of muscle, as has TNF‐related weak inducer of apoptosis (TWEAK), a member of the TNF ligand family [18]. Systemic inflammation characterised by elevated levels of archetypal cytokines is associated with insulin resistance and the development of ICU‐AW [19]. As insulin is an anabolic hormone, resistance will promote a catabolic state. Selenium is an essential trace element that is a constituent of selenoproteins, many of which protect against the effects of oxidative stress. Selenium deficiency has been associated with myopathies [20] and could plausibly exacerbate myopathy.

We hypothesised that major cancer resection surgery was associated with acute reductions in the cross‐sectional area of the RF, of hand‐grip strength, of diaphragm strength and of physical activity. We also aimed to explore (i) the role of insulin resistance and selenium deficiency as potential mediators of these alterations; (ii) the relationship between measures of muscle strength and the functional measure of 6‐min walking distance (6MWD) at Day 7; (iii) the relationship between a measure of physical activity (actigraphy count in the first 48 h following surgery) and the extent of loss of muscle dimensions in the first 7 days.

## 2 Methods

### 2.1 Study Design and Patient Enrolment

The study was conducted at a single NHS Hospital—the Royal Surrey County Hospital, Guildford, UK. Inclusion criteria were age ≥ 18 years and planned for elective open major abdominal surgery for cancer (hepato‐pancreato‐biliary or gynaecological) with predicted duration of surgery of ≥ 3 h. Exclusion criteria were pre‐existing neuromuscular condition, preoperatively invasively ventilated, recent (less than 1 month ago) surgery other than for the purposes of diagnostic staging, and unlikely to survive 1 month postoperatively.

On the day of surgery, preoperative HGS, sniff nasal inspiratory pressure (SNIP) and an ultrasound scan of the right RF were performed. There was a standard operating procedure for each assessment, and all assessments were undertaken by a single trained operator (G.D.) who had demonstrated reliability of repeated measurements (details and Bland–Altman plot in Figure S3). HGS was taken on both hands in triplicate using a hydraulic hand dynamometer (Jamar, USA), and the mean strength was recorded in kg force. SNIP was acquired as in the user manual of the MicroRPM respiratory pressure meter (CareFusion Ltd, UK), and the best reading was recorded in cmH_2_O. The ultrasound imaging of the right RF was done as previously described [6] using the SonoSite X‐Porte ultrasound machine and the C60x 5–2 MHz transducer probe, and three images were acquired. RFCSA was measured using Image‐J software (National Institutes of Health, USA), and the means were recorded in mm^2^. These three assessments were also conducted at Day 7 or day‐of‐discharge (whichever was sooner) and at follow‐up of approximately 6 weeks after hospital discharge.

A MotionWatch 8 (CamNtech Ltd, UK) accelerometer device was fitted to an ankle of each participant on admission to the Intensive Care Unit. The data were recorded in 1‐min epochs in tri‐axial mode. The accelerometer was removed on postoperative Day 7 or day of discharge. Recording and acquisition of activity counts was done using the Actiwatch (Cambridge Neurotechnology Ltd, UK). Early mobilisation is emphasised in most enhanced recovery pathways therefore we used the sum of actigraphy counts during the first two postoperative days.

Routine clinical care of these patients includes early postoperative care taking place within the critical care environment. An electronic patient record (ICCA, Philips) was the source data for all clinical parameters. Clinical parameters, length of ICU stay and hospital stay or ARISCAT, APACHE II or ICNARC scores and outcomes as postoperative morbidity scores (POMS) and postoperative pulmonary complication (POPC) were collected. Functional assessments included the 6MWD test and the Medical Research Council sum score (MRC‐SS). A 6MWD test on Day 7 or on their day of discharge was performed. The distance covered in meters, any stops, breathlessness before and after, and vital parameters (heart rate, oxygen saturation and blood pressure) were recorded.

### 2.2 Biological Sample Handling and Assays

Blood samples were obtained before and 7 days after surgery or on hospital discharge. Plasma levels of cytokines (GM‐CSF, IL‐1β, IL‐6, IL‐8 and TNF‐α) muscle metabolism markers (myostatin, GDF‐15 and IGF‐1), TWEAK, plasma and erythrocyte selenium levels, glucose and insulin levels were measured. Urine was collected for 24 h postoperatively to measure cortisol. Insulin resistance was defined as HOMA2‐IR > 1.39, calculated as the reference value (0.95) plus two standard deviations (0.44). Patients were dichotomised based on postoperative plasma selenium levels, with selenium deficiency defined as < 0.75 μmol/L.

### 2.3 Hypothesis, Sample Size, Primary Outcome and Statistical Analysis

Our sample size calculation was based on a previous study [17] that used a 10% reduction in RFCSA to define clinically meaningful muscle wasting as our primary outcome measure. Using a paired *t*‐test for percentage mean change in muscle cross‐sectional area, a total of 52 participants with two subgroups would give 90% power with alpha level of 5%. Having 25 participants in each surgical group (hepato‐pancreatico‐biliary and gynae‐oncology) would provide 80% power for the subgroups. Data were analysed using PRISM 9 (version 9.3.1 GraphPad Software) in accordance with our prespecified statistical analysis plan as per the study protocol. Data were assessed for normality using visual inspection of histograms and the Kolmogorov–Smirnov test. Normally distributed data were presented as mean (SD) and paired comparisons were ANOVA with Dunnett's correction for multiple comparisons, or paired *t*‐tests if two comparisons were made. If unpaired and two groups then the unpaired *t*‐test was used. Results for nonnormally distributed data were presented as median (IQR), and paired comparisons were Friedman test with Dunn's correction for multiple comparisons, or Wilcoxon test if two paired comparisons were made or Mann–Whitney test if two unpaired comparisons were made. Pearson's or Spearman's correlation coefficients were calculated as appropriate.

## 3 Results

### 3.1 Patients and Data Collection

**FIGURE 1:**
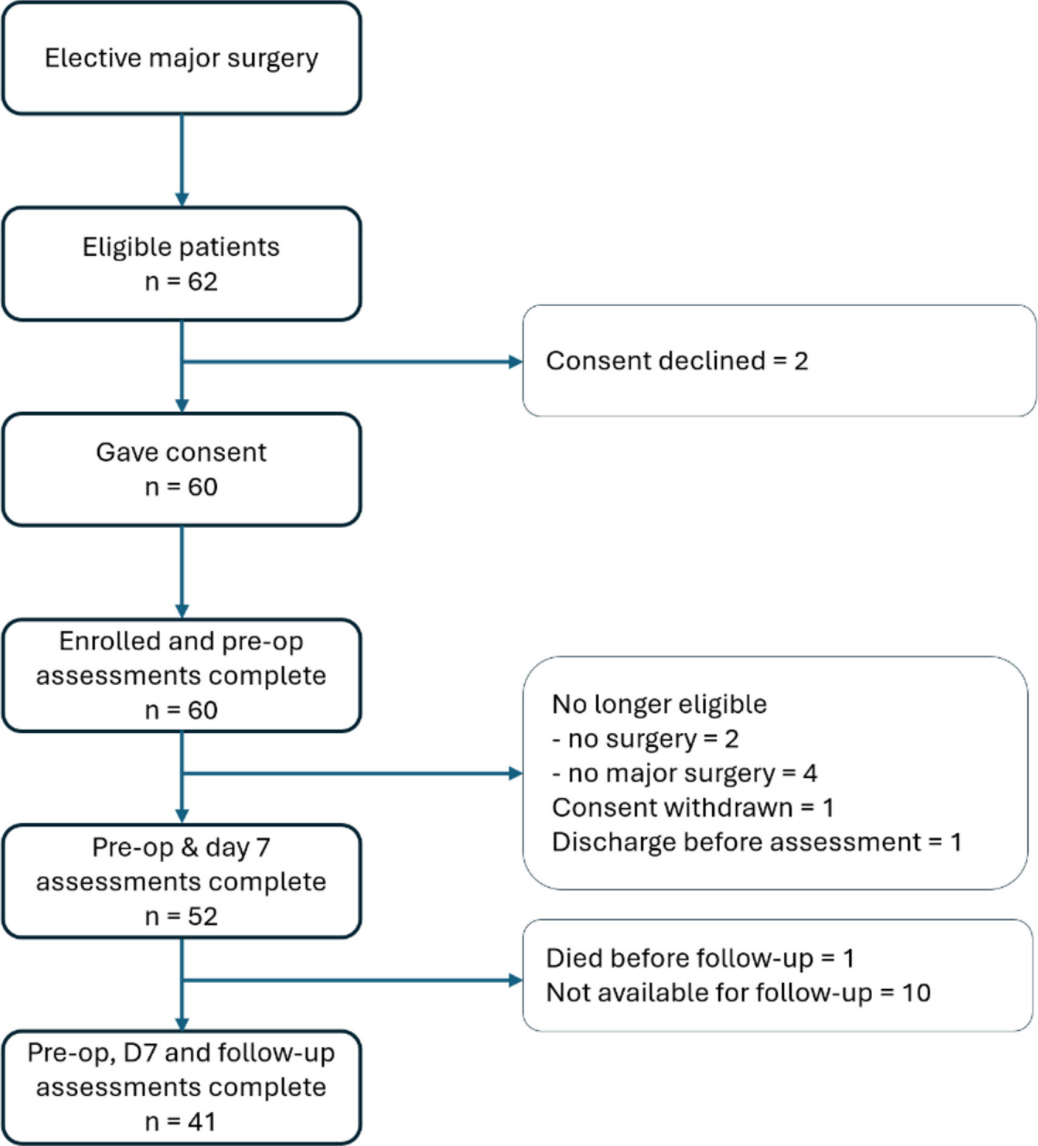
CONSORT diagram for the MAMAS study.

The study recruitment ran for 34 months. Suitable patients were identified and approached; 62 were invited to participate, 2 declined and 60 enrolled. A total of eight participants were withdrawn from the study; four patients did not have major open surgery therefore no longer met the inclusion criteria, two cases got cancelled, one withdrew consent and one was discharged home before the Day 7 study assessments could be completed. For CONSORT diagram see Figure 1.

Fifty‐two participants completed preoperative and postoperative assessments. The elapsed time after surgery for the planned Day 7 assessments was a median of 7 days (IQR 6–7). Forty‐one participants had their 6‐week follow‐up assessments at a median of 57 days (IQR 46–71). Patients' characteristics are summarised in Table 1.

**TABLE 1:** Characteristics of participants: preoperatively, intra‐operatively and duration of stay in ICU and hospital.

	*n* = 52
Age mean (SD) years	64.7 (10.9)
Gender male/female	17/35 (33%/67%)
BMI mean (SD) kg/m^2^	26.1 (4.9)
ASA classification %	
1	6%
2	66%
3	25%
4	3%
Type of surgery	
Gynae‐oncology	26
Hepato‐pancreatico‐biliary	25
Oesophago‐gastric	1
Type of anaesthesia	
GA	2
GA + spinal	2
GA + epidural	48
Duration of surgery median (IQR) minute	259(152)
Duration of anaesthesia median (IQR) minutes	351(144)
Intraoperative blood loss median (IQR) ml	770(850)
Level of ICU admission	
1	0
2	48
3	4
ARISCAT score median (IQR)	41(0)
APACHE II score mean (SD)/median (IQR)	11.6(3.3)/12(5)
ICNARC score mean (SD)/median (IQR)	11.5(4.1)/11.5(4)
Length of ICU stay median (IQR) hours	42.5(45.7)
Length of hospital stay median (IQR) days	7 (5.8)

Abbreviations: APACHE II, Acute Physiology and Chronic Health Evaluation two score; ARISCAT, Assess Respiratory Risk in Surgical Patients in Catalonia (risk index in predicting pulmonary complications after surgery); GA, general anaesthesia; ICNARC, Intensive Care National Audit and Research Centre; ICU, intensive care unit; IQR, interquartile range BMI, body mass index; SD, standard deviation.

### 3.2 Primary Outcome

**FIGURE 2:**
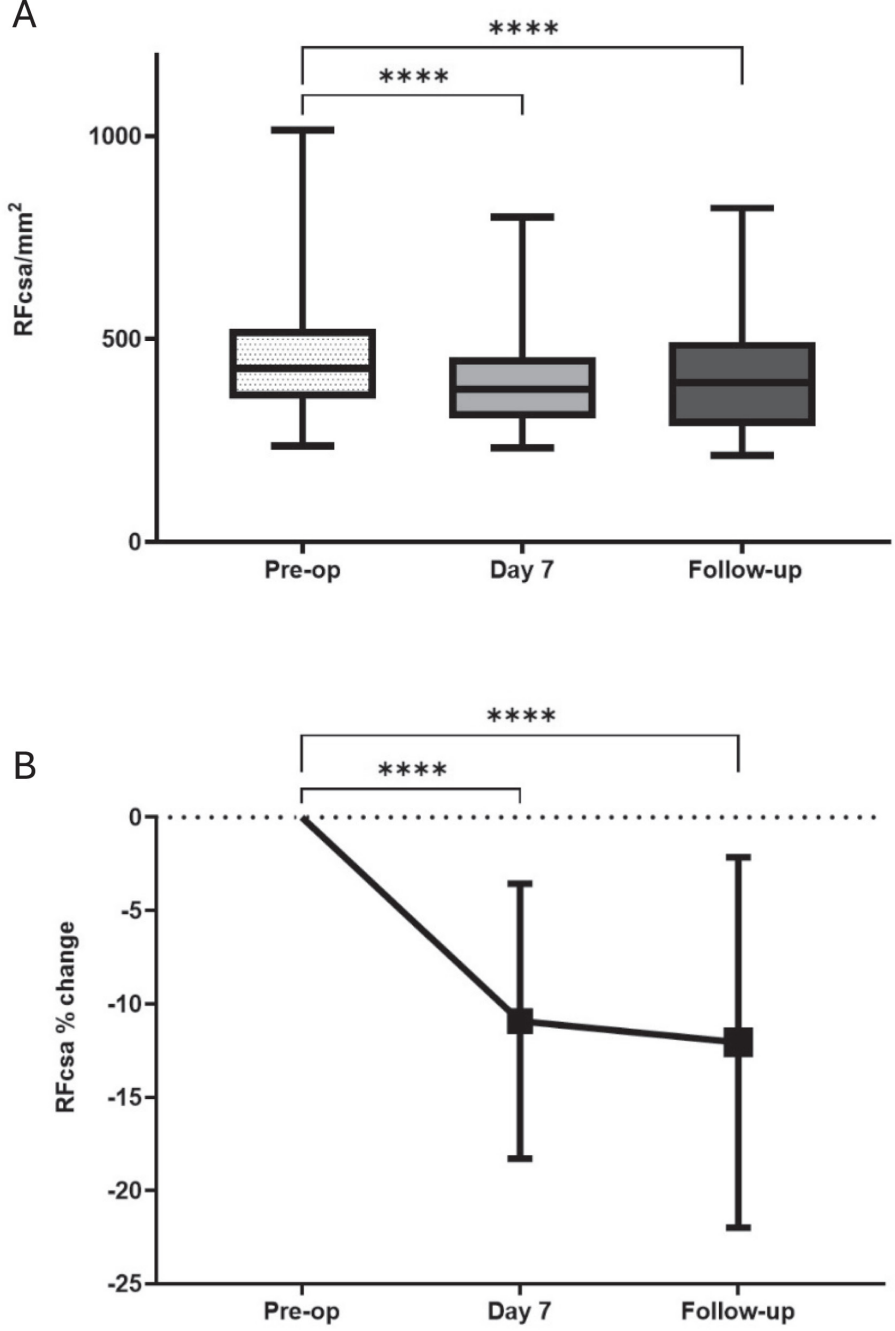
Changes in muscle bulk, rectus femoris cross sectional area (RFCSA) following surgery. (A) Box‐and‐whisker plot (median, IQR, range) of rectus femoris cross sectional area (RFCSA) preoperatively, at Day 7 or discharge, and at follow‐up, *n* = 41. ****p < 0.001 Friedman test with Dunn's correction. (B) Relative change (mean and standard deviation) in RFCSA % change at Day 7 or discharge postoperatively and at follow‐up, *n* = 41. ****p < 0.0001 ANOVA with Dunnett's multiple comparisons test.

The median preoperative and postoperative RFCSA was 383 mm^2^ (IQR 331–513) and 343.7 mm^2^ (IQR 289–451), respectively (*n* = 52). The median of differences in preoperative and postoperative RFCSA was −39.2 mm^2^ (*n* = 52, Wilcoxon test, *p* < 0.0001). This was a −10.2% (SD 7.1, 95% CI −12.1 to −8.2) mean change in muscle size (*n* = 52). We detected a significant change (> 10%, acute myopenia) of RFCSA in 26 (50%) patients. RFCSA decreased significantly from baseline, with median reductions of 50.3 mm^2^ at Day 7 and 35.3 mm^2^ at 6 weeks postoperatively (*n* = 41, *p* < 0.0001, Figure 2A). This corresponded to mean percentage decreases of 10.9% (SD 7.36, 95% CI −13.56 to −8.28) at Day 7 and 12% (SD 9.92, 95% CI −15.62 to −8.51) at 6 weeks (*n* = 41, *p* < 0.0001, Figure 2B).

### 3.3 Secondary Outcomes

**FIGURE 3:**
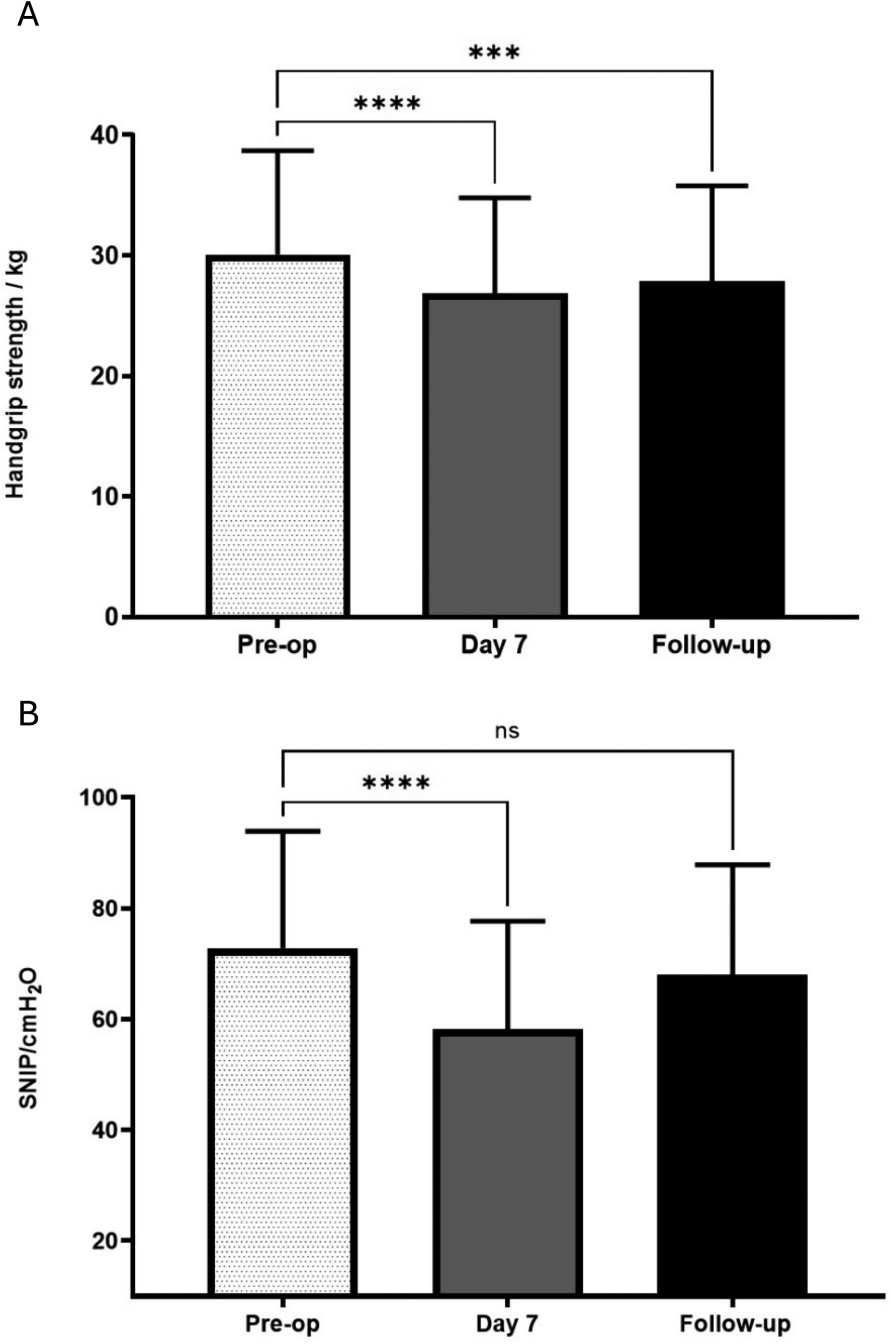
Change in muscle strength, handgrip and sniff nasal inspiratory pressure following surgery. (A) Handgrip strength (HGS) preoperatively, at Day 7 or discharge, and at follow‐up (mean and standard deviation, *n* = 39. ***p < 0.0005 **** *p* < 0.0001 ANOVA with Bonferroni's correction). (B) Sniff nasal inspiratory pressure (SNIP) preoperatively, at Day 7 or discharge, and at follow‐up (mean and standard deviation, *n* = 38. ****p < 0.0001 ANOVA with Bonferroni's correction).

HGS decreased significantly from a preoperative mean of 29.0 kg (SD 8.7) to 25.9 kg (SD 7.9) postoperatively (mean reduction 3.1 kg, SD 2.9, *n* = 50, *p* < 0.0001). At follow‐up (*n* = 39), right‐sided HGS remained below baseline by 3.2 kg (95% CI 2.3–4.1) at Day 7 and 2.3 kg (95% CI 1.05–3.5) at 6 weeks (*p* < 0.0001, Figure 3A).

SNIP decreased significantly from a preoperative mean of 73.4 cmH_2_O (SD 20.9) to 57.5 cmH_2_O (SD 20.8) at Day 7 (mean reduction 15.9 cmH_2_O, SD 18.5, 95% CI −21.2 to −10.6, *n* = 49, *p* < 0.0001). At follow‐up (*n* = 38), SNIP remained 14.6 cmH_2_O (95% CI 8.4–20.7) below baseline at Day 7 and 4.8 cmH_2_O (95% CI −0.5 to 10.0, not significant) below baseline at 6 weeks (*p* < 0.0001, Figure 3B).

**FIGURE 4:**
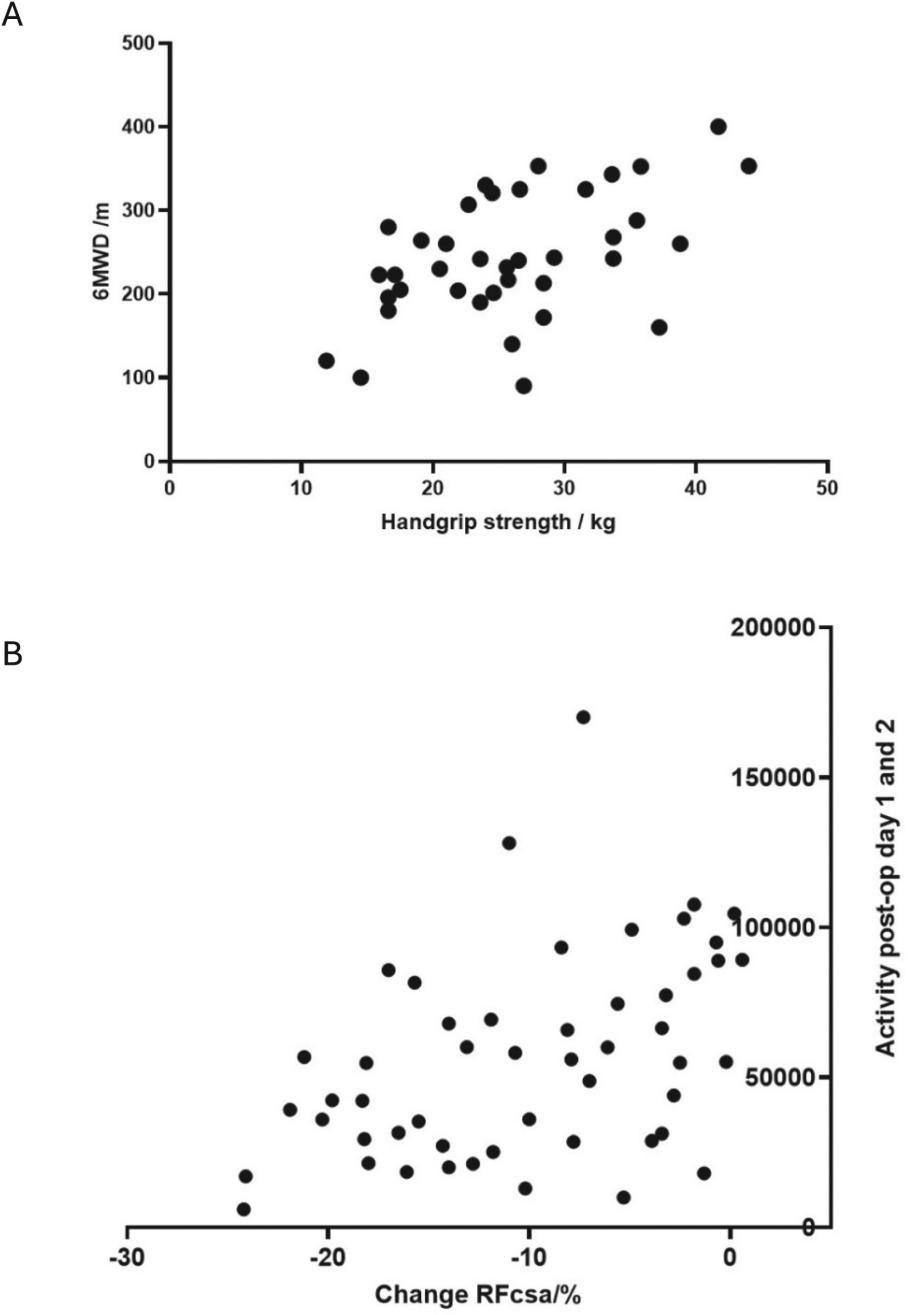
Scatterplots showing relationships between (A)handgrip strength (right) in (kilograms) measured at Day 7 or day of discharge [*x*] and distance walked in 6 min (metres) [*y*] when measured on the same day, *n* = 38. Pearson's *r* = 0.51, *p* = 0.001. (B) Percentage change in rectus femoris cross sectional area (RFCSA) between Day 7 or day of discharge and preop [*x*] and sum of activity counts in first 48 h postop [*y*], *n* = 51. Spearman's *r* = 0.45, *p* = 0.001.

The mean 6MWD at Day 7/discharge was 244.6 m (SD 74.4, *n* = 38). 6MWD correlated positively with HGS (*r* = 0.50, *p* = 0.0011, Figure 4A) but showed no significant association with SNIP (*r* = 0.19, *p* = 0.2497).

Actigraphy showed mean activity counts of 55 962 (SD 33862) during the first two postoperative days. Patients with < 10% RFCSA reduction demonstrated significantly higher activity (68 849 counts) than those with >10% reduction (43 490 counts, mean difference −25 359, 95% CI −43 292 to −7426, *p* < 0.01). Postoperative activity levels positively correlated with RFCSA preservation (*r* = 0.45, *p* = 0.001, Figure 4B).

### 3.4 Clinical Outcomes

Patients with minor complications (POMS 0–2) showed less RFCSA reduction than those with severe complications (POMS 3–4): −9.1% versus −13.3% (mean difference −4.2%, 95% CI −8.6 to 0.2, *p* = 0.063). Patients with and without postoperative pulmonary complications showed similar RFCSA changes: −10.6% versus −9.9% (mean difference −0.68%, 95% CI −5.4 to 4.0, *p* > 0.05). ICU stay duration was comparable between patients with acute myopenia and those without: 41 (IQR 21–66) versus 44 (IQR 34–139) hours (median difference 3.0, 95% CI −27.0‐12.5, p > 0.05). Hospital stay duration was also similar: 7 (IQR 6–14) versus 8 (6–11) days (median difference −0.5, 95% CI −1.5 to 3.0, *p* > 0.05).

### 3.5 Exploratory Biomarker Assays

**FIGURE 5:**
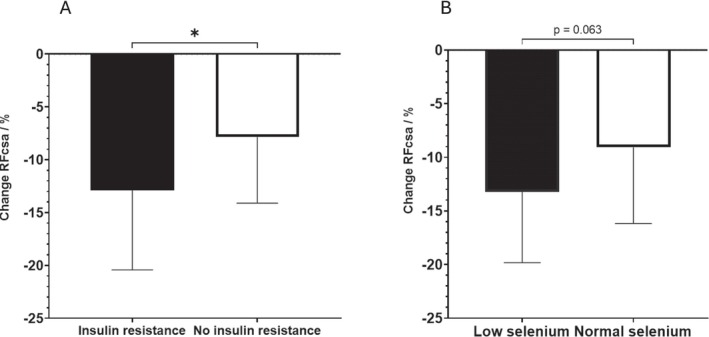
Effect of metabolic parameters on postoperative muscle wasting. (A) Change in rectus femoris CSA (%: mean and standard deviation) in those who had insulin resistance (HOMA2 score > 1.39), and those who did not. *Unpaired *t*‐test, *p* = 0.017, *n* = 46. (B) Change in rectus femoris CSA (%: mean and standard deviation) in those who had low postoperative selenium (plasma selenium < 0.75 μmol/L), and those who did not. **Unpaired *t*‐test, *p* = 0.063, *n* = 51.

Preoperative assays (*n* = 46, 4 patients excluded as on insulin pump, 2 sample errors) showed median serum glucose of 5.9 mmol/L (IQR 5.5–6.5) and insulin of 64.5 pmol/L (IQR 51.3–93.0). Median HOMA2‐IR was 1.27 (IQR 0.98–1.90). Patients with insulin resistance demonstrated significantly greater RFCSA reduction compared with those without: −12.9% (SD 7.52) versus −7.85% (SD 6.26) (mean difference 5.01%, 95% CI 0.95–9.14, *p* = 0.017). See Figure 5A.

Mean plasma selenium decreased significantly after surgery: 0.95 μmol/L (SD 0.17) to 0.84 μmol/L (SD 0.17) (mean difference −0.10, 95% CI −0.14 to −0.06, *p* < 0.0001). Using a plasma selenium of < 0.75 μmol/L to define an abnormally low level, the cohort was dichotomised according to their postoperative plasma selenium levels. Patients with low selenium showed greater RFCSA reduction than those with maintained levels: −13.2% (SD 6.6) versus −9.1% (SD 7.1) (mean difference 3.43, 95% CI −0.54 to 7.42, *p* = 0.06). See Figure 5B. Erythrocyte selenium levels remained stable: 5.83 (SD 1.65) nmol/g Hb before surgery versus 5.84 (SD 1.87) nmol/g Hb after (mean difference 0.01, 95% CI −0.63 to 0.66, *p* > 0.05).

### 3.6 Exploratory Biomarker Assays

Preoperative level of serum glucose was a median 5.9 (IQR 5.5–6.5) mmol/L, serum insulin was a median 64.5 (IQR 51.3–93.0) pmol/L, (*n* = 46, 4 patients excluded as on insulin pump, 2 sample errors). The calculated insulin resistance (HOMA2‐IR [21]) had a median of 1.27 (IQR 0.98–1.90). The mean percentage change of RFCSA was −12.9% (SD 7.52) if a patient had insulin resistance (if HOMA2IR > reference value + 2SD = 0.95 + 0.44 = 1.39) and −7.85% (SD 6.26) if there was no insulin resistance (*n* = 46, unpaired *t*‐test, *p* = 0.017, mean difference 5.01%, 95% CI 0.95–9.14), see Figure 5A.

The mean plasma selenium level before surgery was 0.95 μmol/L (SD 0.17) and was 0.84 μmol/L (SD 0.17) after surgery, with a mean difference of −0.10 (paired *t*‐test, *p* < 0.0001, 95% CI −0.14 to −0.06). Using a plasma selenium of < 0.75 μmol/L to define an abnormally low level, the cohort was dichotomised according to their postoperative plasma selenium levels. Those with a low selenium had a mean % change in RFCSA of −13.2 (6.6) versus −9.1 (7.1) in those who maintained their plasma selenium levels, unpaired *t*‐test *p* = 0.06, mean difference 3.43, 95% CI −0.54 to 7.42, see Figure 5B. The mean erythrocyte selenium level before surgery was 5.83 (SD 1.65) nmol/g Hb and 5.84 (SD 1.87) nmol/g Hb after surgery, paired *t*‐test *p* > 0.05, mean difference 0.01 95% CI −0.63 to 0.66.

**FIGURE 6:**
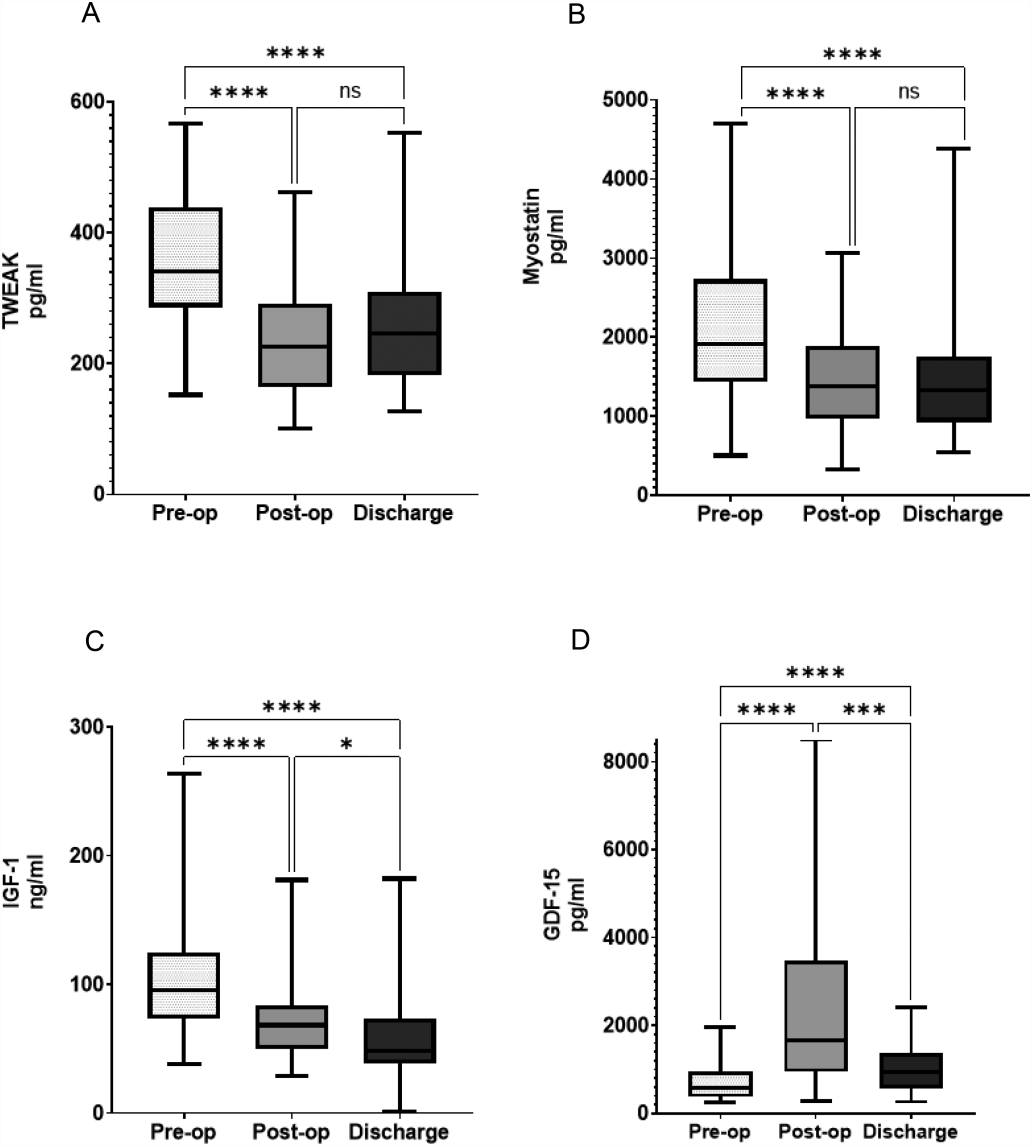
Plasma levels of selected proposed determinants of muscle wasting associated with disease comparing preop, postop and at discharge. (A) Tumour necrosis factor‐related weak inducer of apoptosis (TWEAK). (B) Myostatin. (C) Insulin‐like growth factor‐1 (IGF‐1). (D) Growth and differentiation factor‐15 (GDF‐15). All data represented as median, IQR, range (*N* = 48–51, Friedman test with Dunn's correction ****p < 0.001, ***p < 0.001, *p < 0.05).

Biomarkers that have been associated with muscle wasting included TWEAK, myostatin, IGF‐1 were all decreased and remained so at follow‐up, see Figure 6A–C, respectively. GDF‐15, a biomarker for muscle catabolism, was elevated at Day 7 and remained so at follow‐up, Figure 6D. Biomarkers of systemic inflammation in the early postoperative course included IL‐6, IL‐8, IL‐10 and TNF‐α levels at ICU admission and CRP in the first 2 days and were all elevated, see Figure S1.

## 4 Discussion

Half of our cohort of patients had a significant reduction in the size of the RF muscle in the first week after major cancer surgery. We used the previously described technique for the US scan of the thigh (RFCSA), which is reliable, noninvasive and can be performed at the bedside [22]. The decrease in muscle size did not recover by the time of follow‐up, an average of 2 months later. Consistent with this, there was a significant reduction in HGS and diaphragmatic strength (SNIP) in the early postoperative period. At later follow‐up, the HGS and SNIP remained lower than at baseline. Postoperative daily activity was monitored using an ankle accelerometer and this demonstrated that those who had > 10% loss in muscle size had significantly less activity during the first two postoperative days compared with those who had < 10% loss. There was a moderate positive correlation between activity and % loss of RFCSA. The relationship between postoperative activity and muscle loss is complex, with potential bidirectional effects and confounding factors such as illness severity and pain. Our study design did not allow us to establish a causal relationship or determine the precise temporal sequence between muscle loss and reduced activity. Most (73%) of our participants were able to perform the 6MWD test at the time of discharge; the distance covered was positively associated with HGS.

In those patients who had higher morbidity scores (POMS), there was a greater reduction in RFCSA than in those who had lower morbidity scores; however, this difference was not statistically significant. There was no increased duration of ICU or hospital stay between those with acute myopenia and those without.

Two hypothesis‐generating associations were found between measured biological variables and the occurrence of greater reduction in muscle size (RFCSA): early demonstration of insulin resistance (high HOMA2 scores) and early postoperative depletion of plasma selenium. Insulin resistance has been described in association with cancer‐related cachexia [23], sarcopenia [24] and in experimental models of acute myopenia due to bed rest [25]. In healthy subjects, insulin inhibits proteolysis in a dose‐dependent manner, but in critical illness, it remains to be proven whether insulin therapy can ameliorate protein breakdown [26]. Postoperative depletion of plasma selenium is expected as postoperative systemic inflammation has been associated with depletion of selenoprotein P, an important contributor to plasma selenium [27]. Similarly, hospitalised patients with COVID‐19 were found to be deficient in plasma selenium, along with low concentrations of selenoprotein‐P [28]. The association between low postoperative plasma selenium and increased loss of RFCSA is hypothesis generating, and conclusions about the mechanistic role of selenium deficiency would be premature. Consistent with expectations, circulating levels of inflammatory cytokines (IL‐6, IL‐8, IL‐10 and TNF‐α) were increased immediately postoperatively and C‐reactive protein increased over the subsequent postoperatively days.

Levels of myostatin and IGF‐1 were suppressed after surgery and remained so at discharge. By contrast, levels of GDF‐15 levels were significantly elevated early following surgery and remained elevated compared with baseline at discharge. Reduction in circulating IGF‐1 levels is consistent with reports from other surgical cohorts [15] and may relate to diminished hypothalamic growth‐hormone releasing hormone (GHRH) release, a fall in IGF‐binding protein (IGFBP) or tissue resistance to growth hormone [29]. Myostatin has been described as a myokine with a negative influence on muscle mass, with myostatin inhibitors even proposed as therapies to treat or prevent acute myopenia [30]. Therefore, our findings may appear incongruous with the literature. However, other surgical cohorts have also shown early decreases in circulating levels of myostatin, paired with the rise in GDF‐15 [17]. Levels of TWEAK were suppressed after surgery and remained so at discharge; this has not been previously described.

Strengths of this study include the comprehensive nature of the characterisation, robustly obtained ultrasound and physiological measurements at several standardised times, and detailed biological sampling; the study was prospective and prospectively registered on a publicly available database with a prespecified analysis plan; the types of surgeries were relatively homogeneous. Receipt of early postoperative physical therapy could influence the development of myopenia, and all patients were managed within an enhanced recovery after surgery (ERAS) pathway that focuses specifically on early mobilisation—there was at least daily (often twice daily) receipt of physical therapy.

Nutritional status preoperatively and type and extent of feeding in the early postoperative period may be significant factors that influence the development of myopenia. It is a limitation of this study that we do not have detailed information on these variables. However, the body mass index of our population (mean 26.12 [SD 4.92], range 18.40 to 42, *n* = 52) suggests that only one of the patients was clinically underweight. A recent report in a very similar cohort described how, despite attempting to follow ERAS pathways, delivery of energy and protein was found to be inadequate [31]. The authors recommend involvement of dietitians into the care of these patients. The cohort described in this manuscript all had daily involvement of a specialist dietitian. In a critically ill cohort of patients, increased early delivery of nutrition was not associated with a reduced incidence of ICU‐AW. Perhaps counterintuitively, it was associated with an increased incidence [32].

Limitations include a relatively small sample size, some loss to follow‐up, and the variable influence of the underlying reason for their surgery (cancer) and the potential for bias introduced by the lack of blinding by the outcome assessor. Cancer‐related cachexia is a well‐recognised cause of loss of muscle strength and size. We propose that the changes in muscle homeostasis in our cohort were unrelated to this for the following reasons: (i) our cohort were being optimised for surgery with nutritional support and prehabilitation (exercise) and would not have been selected for major surgery if they were already on a rapidly deteriorating trajectory; (ii) the loss of muscle was very rapid, which is atypical for cancer‐related cachexia; (iii) we have characterised muscle loss after cardiac surgery, which has a similar profile. Our assertion that the surgery causes the acute myopenia would be more robustly demonstrated if patients were randomised to surgery now versus delayed surgery and the rates of change of muscle dimension were compared between the two groups. However, ethical and logistical considerations preclude this. Furthermore, future work should aim to measure muscle dimensions in similar cohorts for several weeks prior to surgery to gather data to support our assertion that the trajectory of muscle loss is substantially different.

Although these patients were all cared for in the ICU these findings are distinct from ICU‐acquired weakness for the following reasons: (i) They were on ICU for routine postoperative monitoring (as is the standard of care in our institution); (ii) they were not invasively mechanically ventilated; (iii) they had a low incidence of receipt of organ support; (iv) their average stay on ICU was short. A significant limitation in interpreting the observed relationships between reduced muscle dimensions and both selenium depletion and insulin resistance is the potential for confounding. These associations may be influenced by unmeasured variables or by each other, as the analyses were unadjusted. The sample size limits adjusted analyses. This confounding risk limits causal inferences, and hence, we describe them as hypothesis‐generating associations.

The generalisability of these findings to *all* patients having surgery is limited as we specifically chose a cohort having prolonged surgery for intra‐abdominal or pelvic cancer. However, similar findings have been described in many other cohorts of patients having surgery. Early work included cohorts having surgery that necessitated the use of cardiopulmonary bypass—cardiothoracic and aortic surgery [17, 33, 34]. Subsequently, in abdominal surgical cohorts, at least four studies prospectively characterised deterioration in muscle dimensions or strength (Table S1a) and at least 10 retrospective analyses compared routinely acquired CT imaging (Table S1b). Meta‐analysis of these results would be hampered because investigators have not used a uniform definition of myopenia, and the time between surgery and postoperative evaluation was highly variable. In some series, the duration of follow‐up was so distant from the time of surgery, and the surgery was for malignancy with a high risk of recurrence, that it may be hard to differentiate acute myopenia from cancer‐related cachexia. Many report associations with adverse outcomes including perioperative complications, cancer recurrence and mortality—however, it is important to be mindful of the possibility that the complications contribute towards the development of myopenia, as opposed to *vice versa*.

Future studies should aim to determine the nadir and longer term trajectory of recovery of the myopenia and the relationship to weakness. Similarly, biological sampling to explore the utility of biomarkers to predict muscle wasting holds promise [35, 36]. Despite the challenges of quantifying preoperative and postoperative nutrition and receipt of physical therapy, these data should be collected systematically. Therapies to prevent or treat ICU‐AW or acute myopenia following major surgery could include pharmacotherapy [37] and neuromuscular electrical stimulation (NMES) [38, 39, 40]. An easily accessible cohort of patients that develops predictable muscle loss could facilitate the development of effective therapies for both ICU‐AW and acute surgical myopenia.

## 5 Conclusions

Acute loss of muscle size in the first week after major abdominal surgery was very common and occurred even in those without complications. At 2 months following surgery, muscle dimensions remained reduced. There was also a significant reduction in HGS and diaphragmatic strength (SNIP). There was an association between a reduction in muscle dimensions with decreased physical activity in the first week postoperatively and functional capacity at hospital discharge.

## Author Contributions

The authors of this manuscript certify that they comply with the ethical guidelines for authorship and publishing in the *Journal of Cachexia, Sarcopenia and Muscle*. All authors made substantial contributions to the conception and design and/or acquisition of data and/or analysis and interpretation of data. All authors participated in the drafting or critical revision of the manuscript and gave final approval of the submitted version. B.C.B. contributed to study conception and design. G.D., M.W., M.R., R.S., A.C. and B.C.B. contributed to acquisition of data. G.D., I.C., M.W., M.R., R.S., A.C., M.G. and B.C.B. contributed to analysis and interpretation of data. G.D., M.G. and B.C.B. drafted the manuscript with G.D., I.C., M.W., M.R., R.S., A.C. and M.G. contributing to critical revision.

## Ethics Statement

This prospective observational cohort study was named Muscle wAsting in Major Abdominal Surgery (MAMAS) and was sponsored by Royal Surrey NHS Foundation Trust. It was registered on the ISRCTN public database (ISRCTN96363642) and received approval from the London South‐East Research Ethics Committee (reference: 15/LO/0139) and have therefore been performed in accordance with the ethical standards laid down in the 1964 Declaration of Helsinki and its later amendments. All persons gave their informed consent prior to their inclusion in the study.

## Conflicts of Interest

The authors declare no conflicts of interest.

## Supporting information


**Figure S1** Inflammatory marker plasma levels.
**Table S1A and B**: Studies of acute myopenia following surgery—muscle dimensions and/or strength.
