# Supplementary material for: Patients Having Major Abdominal Cancer Surgery Exhibit Significant Acute Muscle Wasting
Source: J Cachexia Sarcopenia Muscle. 2025 Jun 13;16(3):e13858. doi: 10.1002/jcsm.13858 (PMC12163515; doi:10.1002/jcsm.13858)
Supplement: Supplementary file 1 — Figure S1 Inflammatory marker plasma levels. Table S1A and B: Studies of acute myopenia following surgery—muscle dimensions and/or strength. [file JCSM-16-e13858-s001.docx]

**Supplemental tables**

**TABLE S1a) Studies of acute myopenia following surgery – muscle dimensions and/or strength. Prospective, chronological.**

| Year | Number of patients | Type of Surgery | Type of study | Measures used | Findings | Times | Reference |
| --- | --- | --- | --- | --- | --- | --- | --- |
| 2017 | 110 | Gastrectomy | Prospective observational | CT imaging. Total abdominal muscle area (TAMA).  Patients with muscle wasting ≥10% were defined to have clinically relevant muscle wasting | - Muscle wasting in 35/110 patients (31.2%). - Association with decreased QoL, physical function (handgrip strength), complications, prolonged hospital stay, higher costs.   Diabetes and advanced age were risk factors | 1 month before versus 1 week after | (1) |
| 2020 | 89 | 65 years and older, elective surgery with at least 60 minutes expected surgical time | Prospective observational (secondary analysis of distinct study) | Handgrip strength, no measurements of muscle dimensions | - Handgrip strength decreased for all patients, at hospital discharge and at 3 months - Lung function   Functional measures | 3 months | (2) |
| 2022 | 15 | Open colorectal resection | Prospective interventional trial | Ultrasound measurements of Vastus Lateralis cross-sectional area (CSA) and muscle thickness (MT) | 9.16% loss of CSA in control limb versus 2.76% loss in the intervention limb | Pre-op and POD5 | (3) |
| 2023 | 173 | Major abdominal | Prospective observational | US of biceps brachii, rectus femoris, and vastus intermedius.  surgery-related muscle loss (SRML) was defined as a decline of 10 per cent or more in diameter in at least one arm and leg muscle within 1 week postoperatively | SRML in 39% | - 1 day pre-op versus 7-days post-op   3, 6 and 12 months | (4) |

**Table S1b) Studies of acute myopenia following surgery – muscle dimensions and/or strength. Retrospective, chronological.**

| Year | Number of patients | Type of Surgery | Measures used | Findings | Times | Reference |
| --- | --- | --- | --- | --- | --- | --- |
| 2016 | 241 | Liver resection surgery for primary HCC | CT imaging of psoas muscle index (PMI) | Decrease in PMI at 7 days, gradually improved by 24 months. | Pre-operatively, 7d, 3m, 6m, 12m and 24m post-op | (5) |
| 2017 | 89 | Radical cystectomy | CT imaging. Changes in total psoas muscle area. Skeletal muscle index (SMI) | Reduction in SMI at 1 month, recovering. | Pre-op and 1-m and 3m post-op | (6) |
| 2017 | 254 | Liver resection surgery | CT imaging. Changes in total psoas muscle area.  Surgery-related muscle loss (SML) was defined as the lowest tertile of percent change in area. | Median % change in total psoas area 7-days after surgery was -2.2% |  | (7) |
| 2018 | 180 | Pancreatectomy | CT imaging. Patients with [muscle wasting](https://www.sciencedirect.com/topics/medicine-and-dentistry/muscle-atrophy) ≥10% of TAMA were defined to have clinically relevant muscle wasting | - Muscle wasting 28.6% in first 60 days after surgery   Associated with worst survival | Pre-operative versus 62 days median time to 2^nd^ CT | (8) |
| 2019 | 316 | Esophagectomy | CT imaging. Skeletal muscle index (SMI) at L3 | - 66% had a >1.25% decrease in SMI   Associated with worst survival | 3m before versus 4 months after surgery | (9) |
| 2019 | 457 | Liver donors | CT imaging of psoas muscle index (PMI) | 4.6-5.0% decrease in PMI at 7 days, improved by 3 months. | 1 week and 3 months | (10) |
| 2020 | 72 | Esophagectomy | CT imaging of psoas muscle area. | - Median of 4.4% loss of psoas muscle area at POD3   Associated with worst survival | Pre-op versus POD3 | (11) |
| 2021 | 128 | Liver resection surgery | CT imaging. Changes in muscle quality and quantity of the psoas muscle. | Loss of quantity in 52% and quality in 65% | Within 6 weeks before versus 3 weeks after surgery | (12) |
| 2022 | 64 | Open versus Laparoscopic liver resection for primary HCC | CT imaging of psoas muscle index (PMI) | - 13.3% of patients had at least 11.5% loss of PMI - Greater extent of loss in open approach versus laparascopic   Metabolomic analysis | Pre-op and around 1m post-op | (13) |
| 2022 | 318 | Gastric cancer | CT imaging of skeletal muscle area indexed to BSA (SMI) | 20.4% had at least 5% loss of SMI | 1 week before versus 6 months after | (14) |

**References**

1. Huang DD, Ji YB, Zhou DL, Li B, Wang SL, Chen XL, et al. Effect of surgery-induced acute muscle wasting on postoperative outcomes and quality of life. Journal of Surgical Research. 2017 Oct 1;218:58–66.

2. Lachmann G, Mörgeli R, Kuenz S, Piper SK, Spies C, Kurpanik M, et al. Perioperatively Acquired Weakness. Anesthesia & Analgesia. 2020 Feb;130(2):341–51.

3. Hardy EJ, Hatt J, Doleman B, Smart TF, Piasecki M, Lund JN, et al. Post-operative electrical muscle stimulation attenuates loss of muscle mass and function following major abdominal surgery in older adults: a split body randomised control trial. Age and Ageing. 2022 Oct 1;51(10):afac234.

4. Hogenbirk RNM, van der Plas WY, Hentzen JEKR, van Wijk L, Wijma AG, Buis CI, et al. Postoperative muscle loss, protein intake, physical activity and outcome associations. British Journal of Surgery. 2023 Feb 1;110(2):183–92.

5. Kobayashi A, Kaido T, Hamaguchi Y, Okumura S, Taura K, Hatano E, et al. Impact of postoperative changes in sarcopenic factors on outcomes after hepatectomy for hepatocellular carcinoma. Journal of Hepato-Biliary-Pancreatic Sciences. 2016;23(1):57–64.

6. Miyake M, Morizawa Y, Hori S, Marugami N, Shimada K, Gotoh D, et al. Clinical impact of postoperative loss in psoas major muscle and nutrition index after radical cystectomy for patients with urothelial carcinoma of the bladder. BMC Cancer. 2017 Mar 31;17:237.

7. Otsuji H, Yokoyama Y, Ebata T, Igami T, Sugawara G, Mizuno T, et al. Surgery-Related Muscle Loss and Its Association with Postoperative Complications After Major Hepatectomy with Extrahepatic Bile Duct Resection. World Journal of Surgery. 2017;41(2):1.

8. Choi MH, Yoon SB, Lee K, Song M, Lee IS, Lee MA, et al. Preoperative sarcopenia and post-operative accelerated muscle loss negatively impact survival after resection of pancreatic cancer. Journal of Cachexia, Sarcopenia and Muscle. 2018;9(2):326–34.

9. Takahashi K, Watanabe M, Kozuki R, Toihata T, Okamura A, Imamura Y, et al. Prognostic Significance of Skeletal Muscle Loss During Early Postoperative Period in Elderly Patients with Esophageal Cancer. Ann Surg Oncol. 2019 Oct 1;26(11):3727–35.

10. Kim YH, Park UJ, Chung HS, Hong SH, Park CS, Choi JH, et al. Recovery of the Psoas Muscle Index in Living Donors after a Right Lobe Hepatectomy for Liver Transplantation: A Single-Center Experience. Transplant Proc. 2019;51(6):1853–60.

11. Maeda N, Shirakawa Y, Tanabe S, Sakurama K, Noma K, Fujiwara T. Skeletal muscle loss in the postoperative acute phase after esophageal cancer surgery as a new prognostic factor. World J Surg Onc. 2020 Jun 26;18(1):143.

12. van Wijk L, van Duinhoven S, Liem MSL, Bouman DE, Viddeleer AR, Klaase JM. Risk factors for surgery-related muscle quantity and muscle quality loss and their impact on outcome. European Journal of Medical Research. 2021 Apr 23;26(1):36.

13. Gau RY, Tsai HI, Yu MC, Chan KM, Lee WC, Wang HE, et al. Laparoscopic liver resection is associated with less significant muscle loss than the conventional open approach. World J Surg Oncol. 2022 Dec 4;20:385.

14. Tan S, Zhuang Q, Zhang Z, Li S, Xu J, Wang J, et al. Postoperative Loss of Skeletal Muscle Mass Predicts Poor Survival After Gastric Cancer Surgery. Front Nutr [Internet]. 2022 Feb 1 [cited 2024 Aug 11];9. Available from: https://www.frontiersin.org/journals/nutrition/articles/10.3389/fnut.2022.794576/full
